# Establishment of Novel Simple Sequence Repeat (SSR) Markers from *Chimonanthus praecox* Transcriptome Data and Their Application in the Identification of Varieties

**DOI:** 10.3390/plants13152131

**Published:** 2024-08-01

**Authors:** Bin Liu, Hua-Feng Wu, Yin-Zhu Cao, Xi-Meng Yang, Shun-Zhao Sui

**Affiliations:** Chongqing Engineering Research Center for Floriculture, Key Laboratory of Agricultural Biosafety and Green Production of Upper Yangtze River (Ministry of Education), College of Horticulture and Landscape Architecture, Southwest University, Chongqing 400715, China; 15705983137@163.com (B.L.); swuwhf@126.com (H.-F.W.); yinzhu202108@163.com (Y.-Z.C.); xm2020@email.swu.edu.cn (X.-M.Y.)

**Keywords:** RNA-seq, EST-SSR, clusterization, polymorphism, identification

## Abstract

*Chimonanthus praecox*, a member of the Calycanthaceae family, is a unique, traditional, and famous flowering economic tree species in China. Despite the existence of several varieties, only a few cultivars have been formally named. Currently, expression sequence tag–simple sequence repeat (EST-SSR) markers are extensively used to identify different species and varieties; a large number of microsatellites can be identified from transcriptome databases. A total of 162,638 unigenes were assembled using RNA-seq; 82,778 unigenes were annotated using the Nr, Nt, Swiss-Prot, Pfam, GO, KOG, and KEGG databases. In total, 13,556 SSR loci were detected from 11,691 unigenes, with trinucleotide repeat motifs being the most abundant among the six repeat motifs. To develop the markers, 64,440 pairs of SSR primers with polymorphism potential were designed, and 75 pairs of primers were randomly selected for amplification. Among these markers, seven pairs produced amplified fragments of the expected size with high polymorphism. Using these markers, 12 *C. praecox* varieties were clustered into two monophyletic clades. Microsatellites in the transcriptome of *C. praecox* exhibit rich types, strong specificity, and great polymorphism potential. These EST-SSR markers serve as molecular technical methods for identifying different varieties of *C. praecox* and facilitate the exploration of a large number of candidate genes associated with important traits.

## 1. Introduction

*Chimonanthus praecox* is commonly known as wintersweet (2n = 22); its unique flowering time and extended blooming period (from November to March) make it a popular perennial ornamental plant in China. Notably, it has a cultivation history of over a thousand years [1,2]. It is native to China, extensively used for cut flowers and as a garden plant, and has been cultivated in the United States, Japan, South Korea, and other countries [3,4]. *C. praecox* detoxifies and treats cough, dizziness, nausea, fever, and rheumatoid arthritis [3,5,6]. There are several cultivated species of *C. praecox*; these species are named and identified based on morphological characteristics, such as petal color or morphology [7,8,9]. However, due to the limited number of morphological features and their susceptibility to environmental factors, employing morphological features to evaluate genetic and phylogenetic relationships may be limited [9].

Molecular markers are powerful tools that can reveal genetic relationships at the DNA level, which is unaffected by environmental factors and exhibits high heritability and easy detection [10]. Various molecular markers have extensively been used in *C. praecox* source conservation and genetic breeding, including random amplified polymorphic DNA (RAPD) [11,12], amplified restriction fragment length polymorphism (AFLP) [13,14], sequence-related amplified polymorphism (SRAP), inter simple sequence repeat (ISSR), and simple sequence repeat (SSR) [4,9,11,12,13,14,15,16,17,18,19]. SSR markers, also known as microsatellites, are co-dominant markers that mainly use tandem repeat sequences of two to five nucleotides as basic repeating units; they can distinguish homozygotes from heterozygotes and detect multiple alleles. In addition, they exhibit rich polymorphisms, are easy to operate, produce reliable results, and exhibit good repeatability. Therefore, they are usually the preferred choice [20]. SSR markers can be developed from genomic and transcriptome databases and are divided into genomic simple sequence repeat (gSSR) and expression sequence tag–simple sequence repeat (EST-SSR) based on the type of data used for their development. The developmental cost of gSSR is relatively high, while EST-SSR markers are relatively cost-effective and exhibit higher cross-species transferability owing to their origin in conserved coding regions [21].

The SSR reaction system for *C. praecox* was established in 2012, marking a significant milestone in the genetic study of this species [14]. Building on this foundation, researchers in 2013 developed SSR molecular markers from the transcriptome database of *C. praecox*, successfully amplifying 17 primer pairs [22]. This breakthrough was complemented by the screening and establishment of 31 EST-SSR markers from *C. praecox* EST sequences, with 8 polymorphic markers selected to analyze genetic diversity and structure across 10 natural populations [23]. The momentum continued in 2014, with an in-depth analysis of SSR distribution characteristics within the *C. praecox* transcriptome database, providing valuable insights into the species’ genetic makeup [9]. By 2018, SSR markers had become instrumental in the authenticity identification of *C. praecox* hybrid progeny, underscoring their practical applications [24]. In 2023, researchers further advanced the field by analyzing the genetic diversity and structure of 69 *C. praecox* samples using 33 SSR molecular markers, revealing crucial data on population genetics [4]. Most recently, in 2024, the genetic diversity of 175 *C. praecox* germplasms was comprehensively analyzed, culminating in the construction of a fingerprint map based on SSR molecular markers. This map represents a pivotal tool for future research and conservation efforts [19]. These advancements provide a robust foundation for the next phase of research, which will focus on translating these genetic insights into practical applications for the breeding and conservation of *C. praecox*. Furthermore, compared with the traditional methods of developing SSR markers, the use of high-throughput sequencing technology enables the efficient development of a large number of microsatellites at a lower cost and effort [25]. Consequently, employing SSR markers represents an efficient approach to identifying *C. praecox* germplasms at the molecular level and genotyping its cultivars. 

In the present study, we sequenced the transcriptome of *C. praecox* using the BGIseq500 platform and assembled 162,638 unigenes. Additionally, we identified SSR loci, designed primer pairs based on these data, and developed and characterized seven novel EST-SSR markers. Furthermore, effective EST-SSR markers were developed from transcriptome sequences to investigate the diversity of different varieties of *C. praecox* and classify varieties.

## 2. Results

### 2.1. Transcriptome Sequencing and Assembly

**Figure 1:**
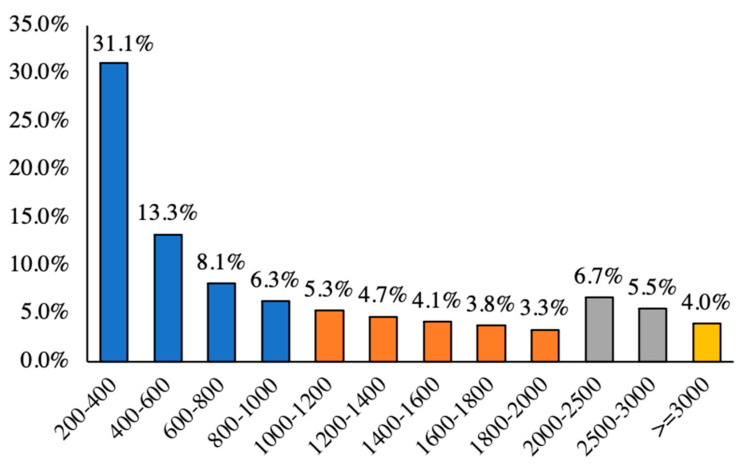
Unigene length distribution information.

A total of 114.73 Gb of clean data were obtained (Table S1), and 162,638 unigenes were assembled. The total length of the unigenes was 170,847,856 bp, and the average length was 1050 bp. Additionally, the GC content was 40.98%, and the N50 was 2059 bp, indicating a high-quality assembly (Table 1). Among them, 80,351 (58.74%) unigenes had a length of 200–1000 bp, 29,042 (21.2%) unigenes had a length of 1–2 kb, 16,654 (12.2%) unigenes had a length of 2–3 kb, and 5449 (4.0%) unigenes had lengths >3 kb (Figure 1).

### 2.2. Functional Annotation

**Figure 2:**
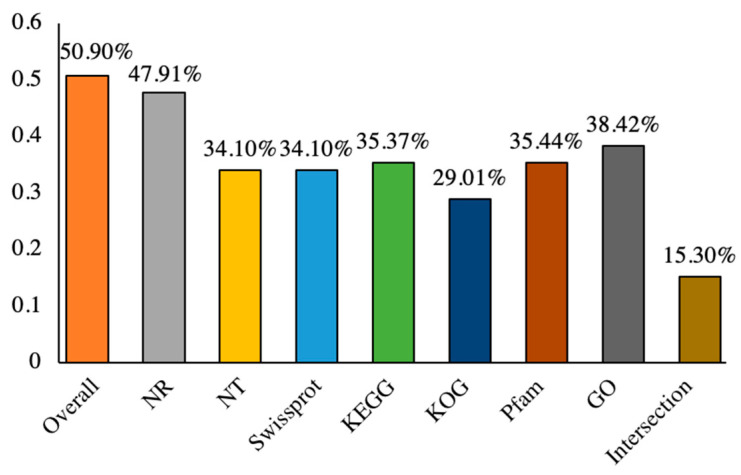
Unigenes annotation of *C. praecox*.

**Figure 3:**
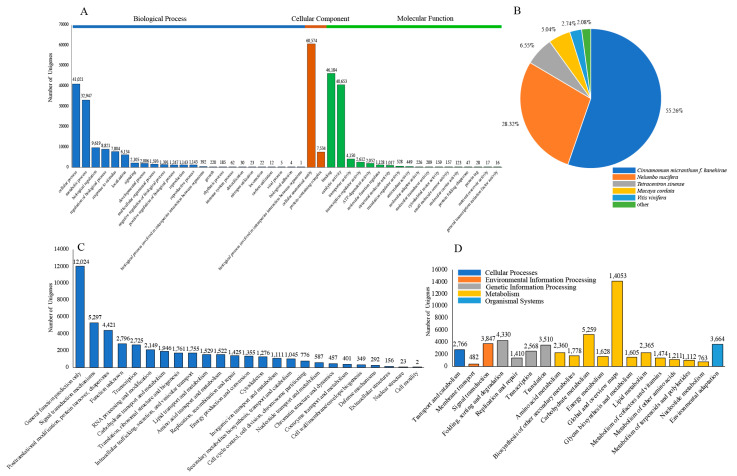
Unigenes annotation based on GO, NR, KOG, and KEGG databases. (**A**) GO annotations of *C. praecox*. (**B**) NR annotations of *C. praecox*. (**C**) KOG annotations of *C. praecox*. (**D**) KEGG annotations of *C. praecox*.

To annotate the unigenes of *C. praecox*, 162,638 single gene sequences were queried against various universal databases. In total, 55,460 (34.10%) were aligned to sequences in the Nt database, 55,465 (34.10%) in the Swiss-Prot database, and 57,638 (35.44%) in the Pfam database (Figure 2). The annotation of 82,778 (50.90%) unigenes was achieved in at least one database, and the annotation of 24,879 (15.30%) unigenes was achieved in all databases (Table 2). A total of 62,480 (38.42%) unigenes were aligned to the sequences in the GO database, which could be divided to three functional categories: biological processes, cellular components, and molecular functions (Figure 2 and Figure 3A). The largest class in biological processes was “cellular processes” (41,021, 25.22%), followed by “metabolic processes” (32,947, 20.26%) and “biological regulation” (9619, 5.91%). The categories of “cellular component” only include “cellular anatomical entity” (60,574, 37.24%) and “protein-containing complex” (7534, 4.63%). Among the molecular functional categories, the largest category was “binding” (46,184, 28.40%), followed by “catalytic activity” (40,653, 25.00%) and “transporter activity” (4150, 2.55%). A total of 77,914 (47.91%) unigenes were aligned to sequences in the Nr database (Figure 2 and Figure 3B). A total of 47,180 (29.01%) unigenes were aligned to sequences in the KOG database, which were categorized into 25 functional groups (Figure 2 and Figure 3C); among them, 12,024 (7.39%) were annotated as “general function prediction only”, followed by “signal transduction mechanisms” (5297, 3.26%), and “posttranslational modification, protein turnover, chaperones” (4421, 2.72%). In total, 56,185 (34.55%) unigenes were aligned to sequences in the KEGG database, which could be categorized into five groups: cellular processes, environmental information processing, genetic information processing, metabolism, and organismal systems (Figure 2 and Figure 3D). Among the 19 biological pathways, the most frequently observed functional pathways were “global and overview maps” (14,053, 8.64%), followed by “carbohydrate metabolism” (5259, 3.23%), and “folding, sorting, and degradation” (4330, 2.66%).

### 2.3. Frequency and Distribution of SSRs in the Transcriptome

**Figure 4:**
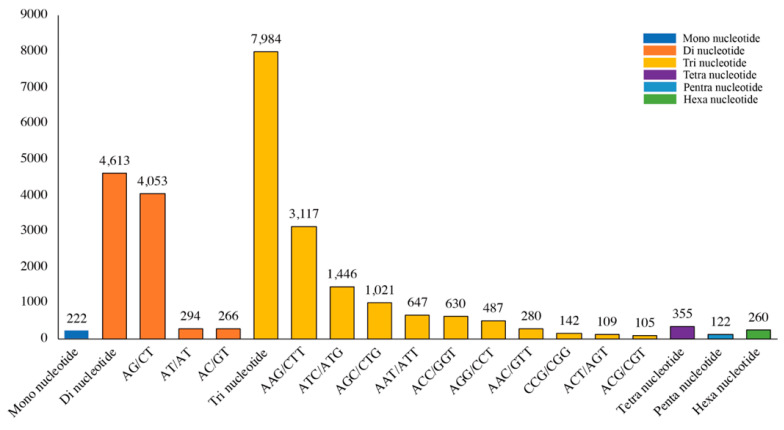
Distribution of microsatellite loci in the transcriptome data of *C. praecox*.

Using the MISA-2.1 software, 13,556 unigenes with a total length of 170,847,856 bp were selected from 162,638 unigenes; 1515 unigenes containing >1 SSR and 11,691 SSR loci were detected (Table 3). Six types of microsatellites were identified from transcriptome data, including mono-, di-, tri-, tetra-, penta-, and hexanucleotide repeat motifs, with significant differences observed among different types of repeat motifs; the trinucleotide repeats exhibited the highest frequency of occurrence (7984, 58.90%), followed by dinucleotides (4613, 34.03%), tetranucleotides (355, 2.62%), hexanucleotides (260, 1.92%), mononucleotides (222, 1.64%), and pentanucleotides (122, 0.90%) (Table 3). The AG/CT (4053) repeats were the most frequent dinucleotide repeats, accounting for 29.90% of the total SSRs. Of the trinucleotide repeats, AAG/CTT (3117, 22.99%) was the most abundant motif, followed by ATC/ATG (1446, 10.67%) and AGC/CTG (1021, 7.53%). The most abundant mononucleotide, tetranucleotide, pentanucleotide, and hexanucleotide repeats were A/T (222, 1.64%), AAAT/ATTT (91, 0.67%), AAAGG/CCTTT or AGCCC/CTGGG (29, 0.21%), and AAGAGG/CCTCTT (33, 0.24%), respectively. The quantities of different dinucleotide and trinucleotide types are shown in Figure 4.

### 2.4. Development of Polymorphic EST-SSR Markers

In total, 75 potential EST-SSR marker primers were designed and validated for polymorphisms in *C. praecox*; 20 of these primers were not amplified, while 55 were successfully amplified, producing amplicons of the expected size. Of the 55 EST-SSR markers, 7 showed high levels of polymorphism and good transferability in different varieties. Genetic variation analysis of the seven loci showed twenty-eight alleles, ranging from two to six, with an average of four alleles per locus. The number of effective alleles (Ne) ranged from 1.492 to 4.235; the total Ne was 20.61, with an average of 2.944. The Shannon’s information index (I) value ranged from 0.512 to 1.585, with an average of 1.122. The observed heterozygosity (Ho) ranged from 0.250 to 1.000. Gene diversity (He) ranged from 0.330 to 0.764, with an average of 0.603. These results indicated that the seven EST-SSR markers had relatively high levels of genetic polymorphisms (Table 4).

### 2.5. UPGMA Cluster Analysis of Different Varieties of C. praecox Based on the EST-SSR Markers

**Figure 5:**
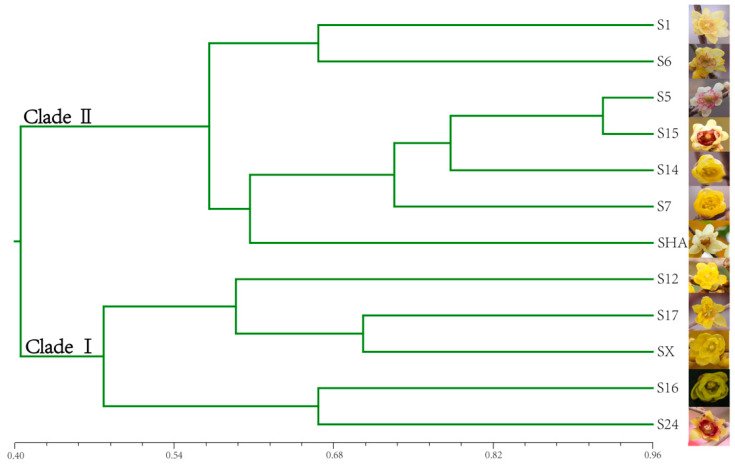
Phylogenetic tree of 12 *C. praecox* varieties generated through UPMGA cluster analysis using the NTSYS-pc 2.0 program. Information on the sample collection of 12 *C.praecox* varieties (S1, S5, S6, S7, S12, S14, S15, S16, S17, S24, SX, and SHA) can be found in Table S2.

A topology tree based on the unweighted pair-group method analysis (UPMGA) was used to display the relationship between the 12 different varieties of *C. praecox* (Figure 5). The r-value of the matrix correlation of the topological tree was 0.808, and the approximate value of the Mantel t-test was 6.13. UPGMA cluster analysis revealed that the 12 varieties of *C. praecox* were clustered into two monophyletic clades; S12, S17, SX, S16, and S24 were clustered in Clade I, and S1, S6, S5, S15, S14, S7, and SHA were clustered in Clade II, indicating close genetic relationships.

## 3. Discussion

*C. praecox*, as an ornamental plant, has been cultivated for more than a thousand years. It originated in China, was introduced in South Korea in the 17th century, and has subsequently been cultivated in other parts of the world such as Japan, Europe, the United States, and Australia [3,4]. After a long history of cultivation, several *C. praecox* varieties have been developed; however, only a few cultivars have been officially named. Notably, among these varieties, there are some homonyms and synonyms [8,26]. Incorrect naming during cultivation has led to difficulties in accurately distinguishing between cultivars [27]. Molecular markers play crucial roles in identifying and characterizing varieties and have been used for variety identification. SSRs, also known as microsatellites, are essential marker systems employed in plant genetic analysis, gene mapping, quantitative trait locus (QTL) mapping, and marker-assisted selection (MAS) breeding due to their high mutation rates, widespread distribution, and high density in a multitude of genomes [28,29]. Notably, their homologous character across related species in DNA coding regions and ample polymorphisms in DNA non-coding regions [28,30] significantly contribute to the large variations observed. SSR markers have been specifically utilized in various identification procedures in several plants, such as *Prunus persica* [31], *Morella rubra* [32], *Punica granatum* [33], and sympodial bamboo [34]. Traditional SSR development methods are difficult, expensive, and labor-intensive; however, next-generation sequencing technology can effectively identify a large number of SSRs at a lower cost with less labor [9,22,23]. Its main advantage lies in its ability to generate a large amount of sequence data, facilitating the isolation and development of a large number of whole genomes and gene-based SSR loci [29,35]. With the advancement in next-generation sequencing (NGS) techniques, new methods of SSR marker development have been discovered; these are grouped into gSSRs distributed throughout the whole genome sequence and EST-SSRs embedded in transcriptional sequences [36,37]. EST-SSRs are more economical compared with gSSRs. Additionally, EST-SSRs demonstrate more efficient amplification, are highly transferable among plant species, and are less susceptible to invalid alleles [10,38]. Transcriptome sequencing has seen recent advancement and is efficient; it enables the discovery of new genes, the identification of gene expression patterns, and the facilitation of the development of molecular markers [39]. In this study, 162,638 unigenes were assembled; the average length of the unigenes was 1050 bp, and the N50 was 2059 bp, indicating the high-quality assembly of transcriptome sequencing data. Transcriptome data provide abundant resources for the SSR sites, which could contribute to the identification and characterization of *C. praecox* varieties. Furthermore, our newly developed microsatellite markers will be useful in the discrimination and identification of *C. praecox* varieties and cultivars.

EST-SSRs are associated with targeted traits that are useful for directing allele selection, detecting functional variations, and analyzing gene-associated genetics [40]. Notably, changes, including replication slippage and other mutational mechanisms affecting SSR, may lead to the gain or loss of function, gene silencing, and the induction of novel proteins, bacterial pathogenesis, or virulence [41]. To obtain a comprehensive functional classification of unigenes in the *C. praecox* transcriptome data, we performed gene function annotations using the public databases of Nr, Nt, Swiss-Prot, Pfam, GO, KOG, and KEGG and found that 50.90% of unigenes were functionally annotated in at least one database, with 15.30% of unigenes functionally annotated across all databases. Additionally, 62,480 (38.42%), 47,180 (29.01%), and 56,185 (34.55%) unigenes were classified into GO, KOG and KEGG categories, respectively; the largest categories in GO, KOG, and KEGG were “cellular processes”, “general function prediction only”, and “global and overview maps” which are valuable for developing functional EST-SSR markers. With the advent of faster and cheaper next-generation DNA sequencing, large amounts of sequence data from different plant species are generated exponentially, and consequently, transcriptome data are being increasingly employed to develop EST-SSR markers [42].

In this study, EST-SSR markers for *C. praecox* were developed using NGS technology. We detected 13,556 EST-SSR loci distributed among 11,691 of 162,638 unigenes. Among the EST-SSR loci, trinucleotide repeat motifs were the most abundant, followed by dinucleotide repeat motifs; this was not consistent with the results of previous research [9,22], which reported that dinucleotide repeat motifs were the most abundant, followed by trinucleotide repeat motifs. However, the controversy associated with *C. praecox* is similar to that reported for *Allium sativum*. Furthermore, Li et al. [21] reported that dinucleotide repeat motifs were the most abundant, which differed from the results of Liu et al. [43], which indicated that trinucleotide repeat motifs were the most abundant. In addition, several plants, including *Elymus sibiricus* [44], *Pueraria thomsonii* [45], *Dolichos bean* [46], *Elymus breviaristatus* [37], and 14 tree species [47], demonstrate a similar pattern, where the trinucleotide repeat is the most abundant in SSR. Variations in previous findings may be attributed to the SSR search criteria, the size of the dataset, and the database mining tools [21].

Microsatellite markers have been extensively used in species and cultivar identification to check the effectiveness of newly developed EST-SSR markers [4,19,24]. Seventy-five pairs of SSR primers were randomly selected to assess the genetic diversity of the genotypes of 12 *C. praecox* varieties. In total, 66.7% of markers successfully amplified target bands, with 9.3% of markers showing high polymorphism. In addition, 33.7% of the markers failed to amplify any fragments, potentially because the primers designed spanned splice sites or large introns within the target amplicon [48]. Using cluster analysis, twelve varieties of *C. praecox* were clustered into two monophyletic clades; seven varieties were clustered in Clade I and five varieties were clustered in Clade II. In most cases, *C. praecox* cultivars were categorized into three groups based on the color of the inner tepals: the Patens, Intermedius, and Concolor groups [2,7,49]. Two varieties of the Intermedius group and two varieties of the Patens group could be classified into one group, and the two Concolor varieties in Clade I demonstrated a close relationship. The two Concolor varieties and one Intermedius variety classified into one group in Clade II showed a close relationship. The results indicated that EST-SSR markers significantly distinguished different varieties based on the inner tepal color. This suggests that the EST-SSR markers may be associated with the flower color phenotype. Notably, SSR may be related to targeted traits and play important roles in development, gene regulation, and evolution [50]. The results of the present study demonstrated that phylogenetic analysis based on EST-SSR markers can provide valuable references for variety identification and reveal a potential connection with the color of inner tepals, providing a premise for the breeding of new varieties of *C. praecox.*

## 4. Materials and Methods

### 4.1. Plant Materials and DNA/RNA Extraction

Twelve different varieties of *C. praecox* plant materials were collected from the resource nursery at the Key Laboratory of Agricultural Biosafety and Green Production of the Upper Yangtze River (Ministry of Education) of Southwest University in Beibei District, Chongqing, in 2023 and used for transcriptome sequencing and the identification of polymorphisms (Table S2). Fresh leaf tissues were cleaned and immediately preserved in liquid nitrogen until DNA and RNA were extracted. Total genomic DNA was extracted from leaves using the CTAB method [51]. Furthermore, two varieties (SHA and SX) were selected for RNA extraction; the RNArep Pure kit (Tiangen Biotechnology, Beijing, China) was used to extract total RNA. To ensure the quality and quantity of the DNA/RNA, 1% agarose gel electrophoresis was used to observe the DNA/RNA extract, and a NanoDrop ND-1000 Spectrophotometer (Thermo Fisher Scientific, Wilmington, MA, USA) was used for quantitative detection.

### 4.2. Transcriptome Sequencing De Novo Assembly

Total RNA samples of acceptable purity and concentration were obtained. Next, library construction was performed, mRNA was enriched using oligo (dT)-attached magnetic beads, and the purified mRNAs were fragmented. First-strand cDNA was synthesized using reverse transcriptase. Furthermore, double-stranded cDNA, synthesized using the first-strand cDNA as a template, was subjected to end-repair of the double-stranded cDNA fragments. Next, a single ‘A’ nucleotide was added to the 3′ ends of the blunt fragments, and adaptor ligation was subsequently configured and set up to ligate adaptors with the cDNAs. The final library was amplified using phi29 DNA polymerase to create DNA nanoballs (DNBs) with over 300 copies of molecules and to check the quality of library construction. The DNBs were loaded into a patterned nanoarray, and a counter terminal reading of 100 base pairs on the BGIseq500 platform (BGIseq500, Shenzhen, China) was generated. Measurement was conducted in triplicate.

### 4.3. Raw Data Analysis and Function Annotation

The raw data were filtered using SOAPnuke (v1.5.2) [52] by first removing reads containing adapters (adapter contamination), reads with an unknown base (‘N’ base) ratio >10%, and reads with a low-quality base ratio (base mass ≤ 15) > 50%; the clean reads stored were stored in FASTA format. After obtaining clean reads and downloading genome data of *C. praecox* (684 Mb in size) from published databases, we used HISAT to align them with the reference genome sequence. Furthermore, the assembled unigenes were annotated with seven major functional databases, including KEGG (Kyoto Encyclopedia of Genes and Genomes), GO (Gene Ontology), NR (National Center for Biotechnology Information nonredundant protein sequences), NT (Nucleotide Sequence Database), Swiss-Prot (Swiss-Prot Sequence Database), Pfam (Protein Families Database), and KOG (EuKaryotic Orthologous Groups of proteins), and the transcription factors were predicted [53,54,55,56,57,58,59].

### 4.4. Microsatellite Identification, PCR Amplification, and Data Analysis

MISA [60] was used to detect microsatellite loci according to the following criteria: mono-nucleotide repeat motif repeat count ≥20, dinucleotide repeat motif repeat count ≥10, and other types of repeat motif repeat counts ≥5. Using Primer3-2.4.0 [61] software to design primers, 75 pairs of primers with target product sizes between 100 and 300 bp were randomly selected. Twelve *C. praecox* varieties were amplified to investigate polymorphisms in the SSR loci. PCR products were visualized using 8% polyacrylamide gel electrophoresis, and SSR was selected to amplify the expected product size to evaluate polymorphisms. The products were placed in gel with 1×Tris-borate-EDTA (TBE) buffer solution and run for 1.5 h at 200 V with 2000 bp molecular size ladder (Tiangen Biotech Co., Ltd., Beijing, China) (Figure S1). Next, the bands were observed using silver staining. For SSR data analysis, alleles were manually scored based on size, with the absence of bands interpreted as “0”, and the presence of bands interpreted as “1”. Genetic information such as the number of alleles (Na), the effective number of alleles (Ne), Shannon’s information index (I), and the Fixed index (F) of each locus was calculated using GenALEX 6.5 [62]. UPMGA cluster analysis was conducted using the NTSYS-pc 2.0 program [63].

## 5. Conclusions

A large number of SSR loci were identified using transcriptome data, and highly polymorphic microsatellite markers were developed and employed to differentiate *C. praecox* varieties. Twelve varieties were categorized into two monophyletic clades. The molecular markers developed in this study will contribute to the identification of *C. praecox* varieties and provide a premise for conducting functional genomic, population genetic, and phylogenetic analyses of *C. praecox.* The above results can provide reference and guidance for functional research on horticultural plants, the identification of different varieties, and molecular breeding.

## Figures and Tables

**Table 1:** Overall data quality and assembly information.

Item	Number
Total clean data (Gb)	114.73
Total unigenes	162,638
Total length of unigenes (bp)	170,847,856
Average length of unigenes (bp)	1050
N50 of unigenes (bp)	2059
GC content	40.98%

**Table 2:** Unigenes annotation of *C. praecox*.

Annotation Database	Number of Unigenes	Percentage (%)
NR	77,914	47.91%
NT	55,460	34.10%
Swiss-Prot	55,465	34.10%
KEGG	57,525	35.37%
KOG	47,180	29.01%
Pfam	57,638	35.44%
GO	62,480	38.42%
Intersection	24,879	15.30%
Overall	82,778	50.90%
Total	162,638	100.00%

**Table 3:** Prediction of SSRs from the transcript datasets of *C. praecox*.

Item	Number
Total number of sequences examined	162,638
Total size of examined sequences (bp)	170,847,856
Total number of identified SSRs	13,556
Number of SSR-containing sequences	11,691
Number of sequences containing more than 1 SSR	1515
Number of SSRs present in compound formation	667
Mononucleotide	222
Dinucleotide	4613
Trinucleotide	7984
Tetranucleotide	355
Pentanucleotide	122
Hexanucleotide	260

**Table 4:** Sequence and genetic diversity information of the seven SSR markers.

Locus	Motif	Forward Primer (5′-3′)	Reverse Primer (5′-3′)	GenBank Accession Number	Na	Ne	I	Ho	He
CP14	(CTT)7	CGCTCTCTCCTTAACGCGAT	ACTTCTTGCTTTTGCCGCTG	PP532794	2.000	1.492	0.512	0.417	0.330
CP20	(TC)25	CCATCTGCGACTGTCCCTTT	CGGATCTCTCCCGGATTTCG	PP532795	4.000	3.646	1.332	0.500	0.726
CP22	(CT)18	AGAACATGTCCAATTCCCATGGA	GCATGCTCGCTCTCTCTCTC	PP532796	6.000	4.235	1.585	0.333	0.764
CP33	(AT)10	CAGTCAGGTCCACGTGTTGA	ATCTCGATCTGCTGCCACTG	PP532797	6.000	3.176	1.426	0.444	0.685
CP43	(GA)14	TGCCCAGTTGCCTCTTTTCA	CGACTTCTTCTCCTTCGCCA	PP532798	2.000	1.492	0.512	0.250	0.330
CP44	(TCG)7	CCGGAAGTAGCCATCGGATC	GCATGGAGAGTCCTCGCTAC	PP532799	3.000	2.969	1.093	0.750	0.663
CP67	(AG)22	CACGAAGCCCTCCAGAAAGT	CTTGCAGGGGAGCATGTACA	PP532800	5.000	3.600	1.393	1.000	0.722

## Data Availability

The clean data of RNA-seq generated in this study have been submitted to the BioProject database of the National Center for Biotechnology Information, number PRJNA1091468.

## Supplementary Materials

The following supporting information can be downloaded at: https://www.mdpi.com/article/10.3390/plants13152131/s1, Figure S1. Amplified profile of markers (Samples were listed in Table S2; L: 2000 bp ladder). (A). Amplified profile of marker CP14 and CP20. (B). Amplified profile of marker CP22 and CP33. (C). Amplified profile of marker CP43 and CP44. (D). Amplified profile of marker CP67. Table S1. Statistical information on the quality of transcriptome sequencing data. Table S2. Sample collection of 12 *Chimonanthus praecox* varieties.
