# Supplementary material for: Establishment of Novel Simple Sequence Repeat (SSR) Markers from Chimonanthus praecox Transcriptome Data and Their Application in the Identification of Varieties
Source: Plants (Basel). 2024 Aug 1;13(15):2131. doi: 10.3390/plants13152131 (PMC11313930; doi:10.3390/plants13152131)
Supplement: Supplementary file 1 [file plants-13-02131-s001.zip › Figure S1. Amplified profile of markers (Samples were listed in Table S2; L- 2000 bp ladder).pdf]

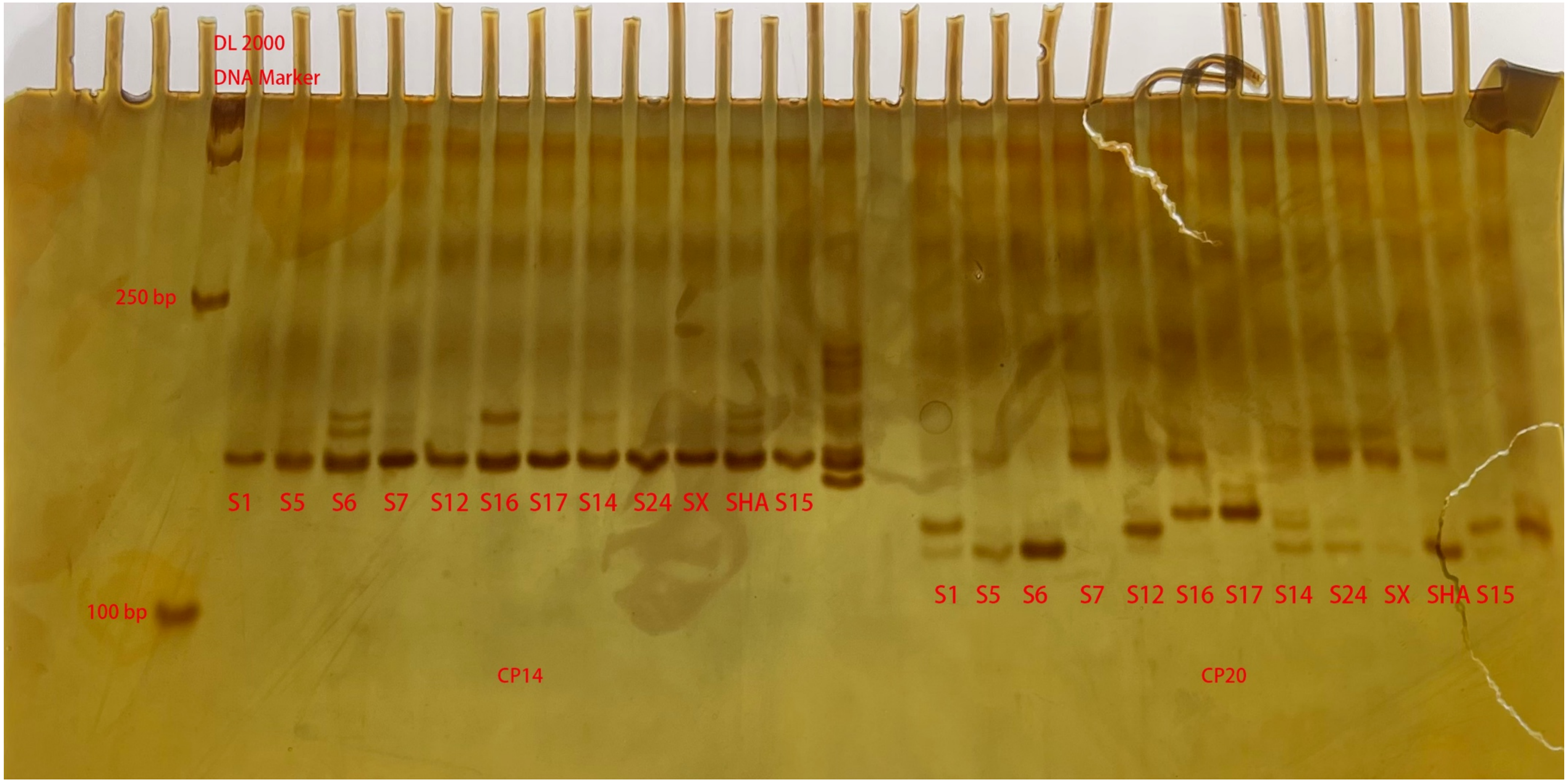
DL 2000
DNA Marker
250 bp
100 bp
S1 S5 S6 S7 S12 S16 S17 S14 S24 SX SHA S15
S1 S5 S6 S7 S12 S16 S17 S14 S24 SX SHA S15
CP14
CP20

A

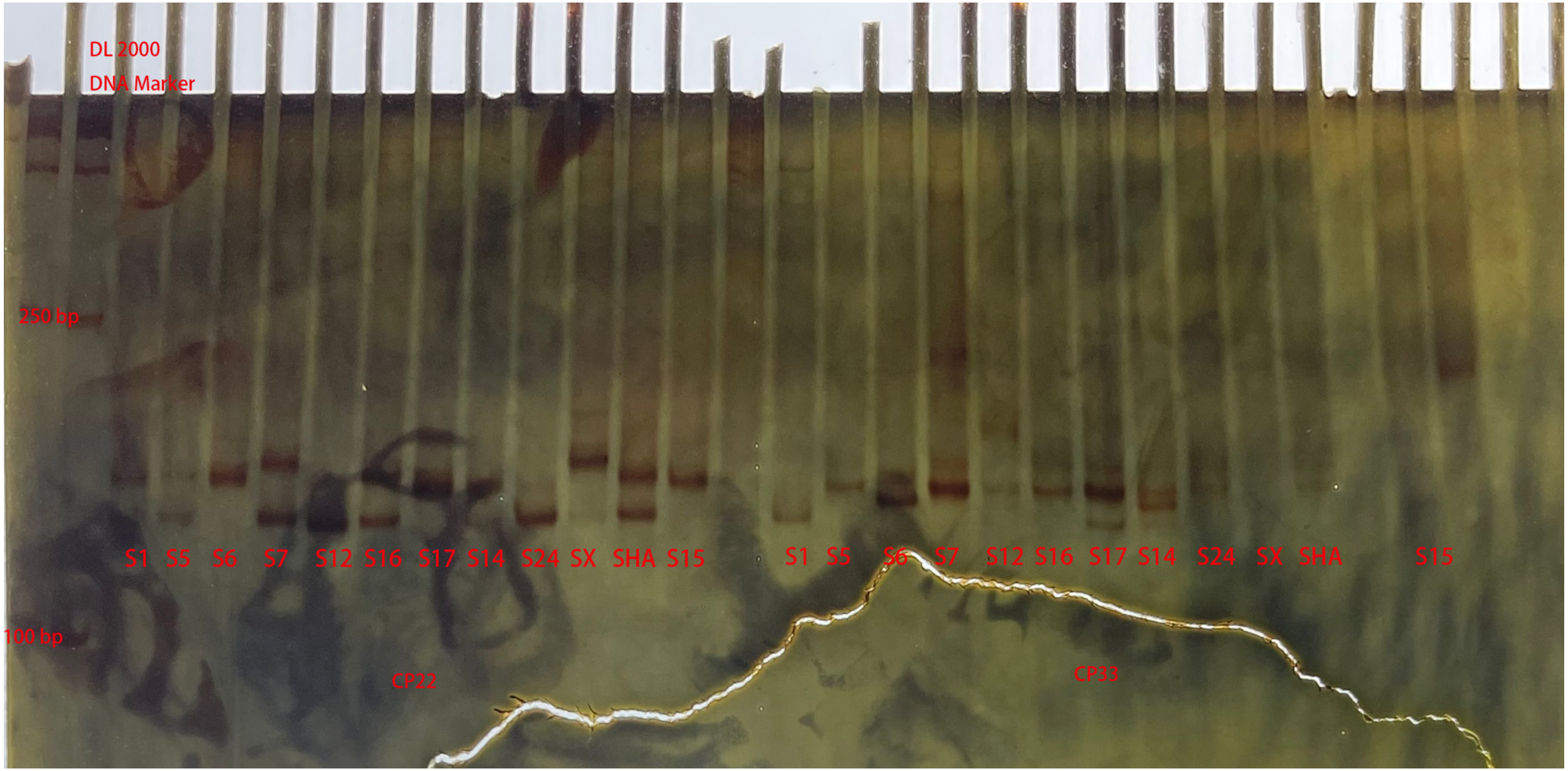
DL 2000
DNA Marker
250 bp
100 bp
S1 S5 S6 S7 S12 S16 S17 S14 S24 SX SHA S15
S1 S5 S6 S7 S12 S16 S17 S14 S24 SX SHA S15
CP22
CP33

B

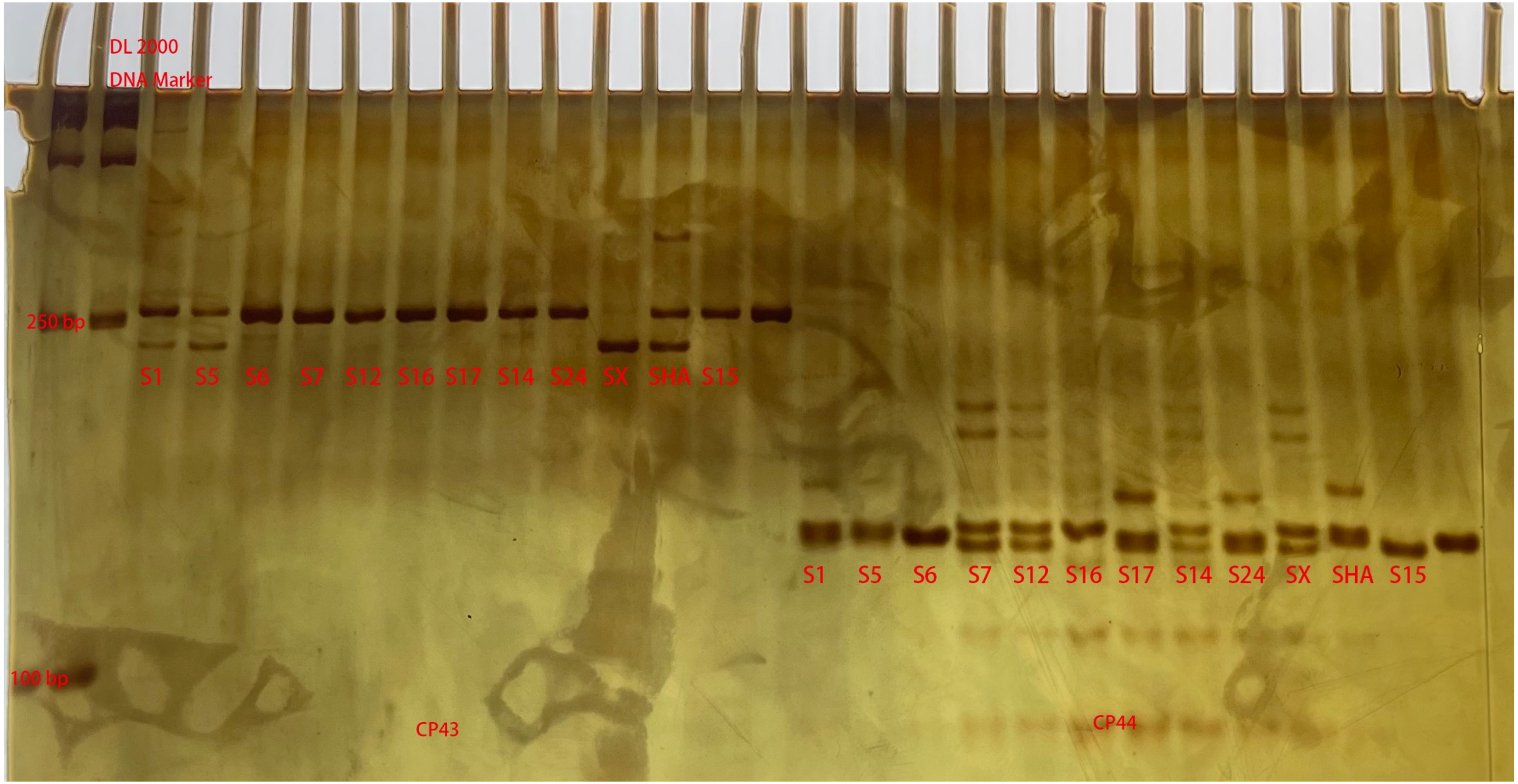
DL 2000
DNA Marker
250 bp
100 bp
S1 S5 S6 S7 S12 S16 S17 S14 S24 SX SHA S15
CP43
S1 S5 S6 S7 S12 S16 S17 S14 S24 SX SHA S15
CP44

C

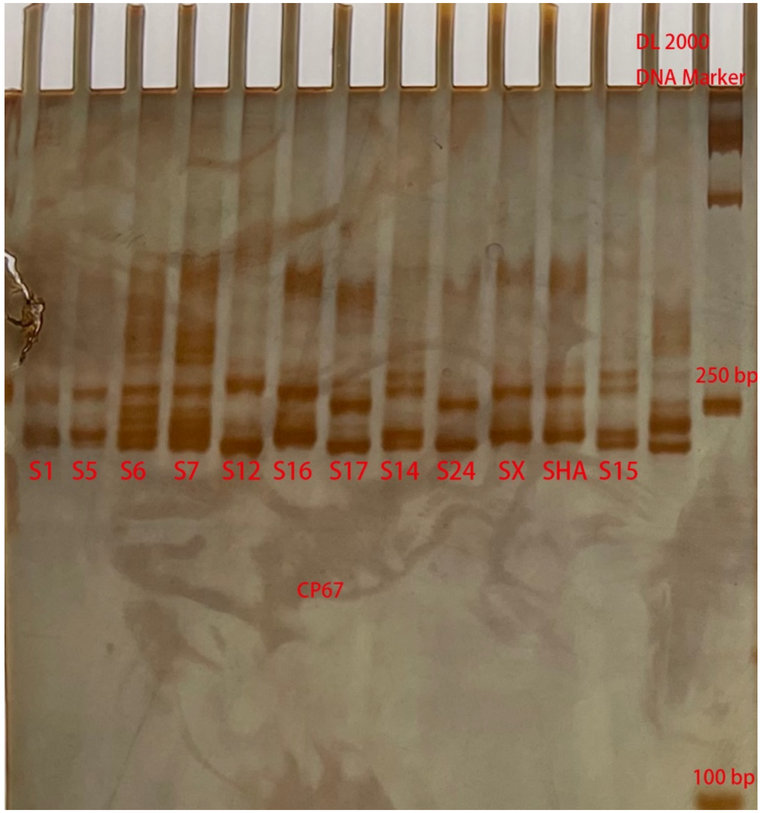

D

Figure S1. Amplified profile of markers (Samples were listed in Table S2; L: 2000 bp ladder). (A). Amplified profile of marker CP14 and CP20. (B). Amplified profile of marker CP22 and CP33. (C). Amplified profile of marker CP43 and CP44. (D). Amplified profile of marker CP67.
